# An Inhibition of p38 Mitogen Activated Protein Kinase Delays the Platelet Storage Lesion

**DOI:** 10.1371/journal.pone.0070732

**Published:** 2013-08-13

**Authors:** Andrey Skripchenko, Helen Awatefe, Dedeene Thompson-Montgomery, Andrew Myrup, Annette Turgeon, Stephen J. Wagner

**Affiliations:** American Red Cross Biomedical Services, Holland Laboratory, Rockville, Maryland, United States of America; University of Leuven, Belgium

## Abstract

**Background and Objectives:**

Platelets during storage undergo diverse alterations collectively known as the platelet storage lesion, including metabolic, morphological, functional and structural changes. Some changes correlate with activation of p38 mitogen activated protein kinase (p38 MAPK). Another MAPK, extracellular signal-related kinase (ERK), is involved in PLT activation. The aim of this study was to compare the properties of platelets stored in plasma in the presence or absence of p38 and ERK MAPK inhibitors.

**Materials and Methods:**

A single Trima apheresis platelet unit (n = 12) was aliquoted into five CLX storage bags. Two aliquots were continuously agitated with or without MAPK inhibitors. Two aliquots were subjected to 48 hours of interruption of agitation with or without MAPK inhibitors. One aliquot contained the same amount of solvent vehicle used to deliver the inhibitor. Platelets were stored at 20–24°C for 7 days and sampled on Days 1, 4, and 7 for 18 in vitro parameters.

**Results:**

Inhibition of p38 MAPK by VX-702 leads to better maintenance of all platelet in vitro storage parameters including platelet mitochondrial function. Accelerated by interruption of agitation, the platelet storage lesion of units stored with VX-702 was diminished to that of platelets stored with continuous agitation. Inhibition of ERK MAPK did not ameliorate decrements in any in vitro platelet properties.

**Conclusion:**

Signaling through p38 MAPK, but not ERK, is associated with platelet deterioration during storage.

## Introduction

In the human body, the natural life span of platelets (PLTs) is between 8 to 10 days. The shelf-life of PLTs collected at blood centers in the United States is limited to 5 days of storage due to the increased risk of bacterial outgrowth to high titers during room temperature PLT storage without pathogen inactivation [1]. Another factor affecting the period of PLT storage is the accumulation of deteriorative changes leading to progressive damage in PLT function and structure which is known as the PLT storage lesion (PSL) [2]. In numerous studies, the PSL is associated with increased glycolysis with decreased pH levels resulting in cytoskeletal reorganization and shape change, reduced aggregation response, secretion of PLT granules, increased production of reactive oxygen species, changes in the lipid membrane, and functional changes that are indicative of apoptosis, such as loss of mitochondrial membrane potential (MMP) and increased phosphatidylserine exposure [3]–[7].

The rapidity of PSL development is influenced by collection methods, post-collection manipulation and storage conditions [8]–[10]. Storage conditions may vary based on PLT count, container size, material used for container manufacture, storage temperature, method of PLT agitation and media used for PLT suspension [11]–[13]. Storage conditions also depend on whether PLTs are shipped. When PLTs are shipped to different destinations, they are packed into shipping boxes and agitation is very limited. Although periods without agitation of 24 hours or less do not diminish PLT in vitro storage parameters, extended periods without agitation, 48 hours and more, accelerate all deteriorative changes which are specific to the PSL of normal storage [10], [14].

The reduction of glycoprotein expression on the PLT surface, in particular GP1bα, the subunit of the GP1b-IX-V complex responsible for the von Willebrand factor interactions, is another characteristic of a progressive decrement associated with the PSL. The loss of GP1bα is negatively correlated with mouse PLT in vivo survival [15]. Shedding of GP1bα is carried out by tumor necrosis factor-alpha-converting enzyme (TACE/ADAM17), matrix metalloproteinase type 1 [15]. Canault and colleagues demonstrated that metalloproteinase 1, TACE, is activated via a p38 mitogen activated protein kinase (MAPK) dependent pathway [16]. Inhibition of p38 MAPK during PLT storage results in a markedly improved posttransfusion PLT recovery in mice, which was correlated with prevention of GP1bα proteolysis [16]. In addition, it has been shown that inhibition of p38 MAPK during PLT storage decreases PLT activation [17]. p38 MAPK is one of the three main classes of MAPKs. The other two classes are c-Jun amino-terminal kinases (JNKs) and extracellular signal-related kinases (ERKs) (ERK1/2). It has been shown that both JUN1 and ERK2 are involved in thrombus formation and have similar up-regulation [18]. Lee and colleagues demonstrated that oxidative damage mediated by ERK1/2 activation induces apoptosis of murine fibrosarcoma cells [19].

The aim of this study was to compare the properties of PLTs stored in 100% plasma with continuous agitation and interruption of agitation for a prolonged time in the presence and absence of p38 and ERK MAPK inhibitors to better identify pathways that may be involved in the PLT storage lesion.

## Materials and Methods

### Ethics Statement

This study was approved by Holland Laboratory Institution Review Board. Human subjects provided IRB approved written informed consent for participation in the study.

### Materials

Dimethylsulfoxide (DMSO) was purchased from Sigma-Aldrich (Sigma-Aldrich Corporation, St. Louis, MO). Inhibitors to p38 MAPK, VX-702 and to ERK, PD98059, were purchased from Selleck Chemicals LLC (Houston, TX).

### PLT collection and study design

A single PLT apheresis unit was collected in 100% plasma from consenting healthy donors using the Trima cell separator (software version 5.1, Terumo BCT Inc., Lakewood, CO) with the targeted yield of 4×10^11^ PLTs. The unit was divided into 5 identical 60-mL aliquots in CLX storage bags (Medsep Corporation, a Subsidiary of Haemonetics Corporation, Covina, CA) immediately after collection. Aliquot A) served as continuous agitation control (Control-CA); aliquot B) was designated as continuously agitated test (Test-CA) with addition of MAPK inhibitor; aliquot C) was allocated as continuous agitation sham control (Sham) and contained the same amount of DMSO as aliquots B and E but without inhibitor; aliquot D) included an interruption of agitation for 48 hours between Day 1 and Day 3 of storage and served as a control to aliquot E (Control-IA); aliquot E) acted as a test with addition of MAPK inhibitor and interruption of agitation (Test-IA) equal to that of aliquot D. With these aliquots, two studies were carried out. In Study I, a second generation p38 MAPK inhibitor, VX-702 was utilized (n = 12). In Study II, six experiments were conducted using ERK MAPK inhibitor, PD98059.

In the IA arm, aliquots D and E were placed in a standard shipping box (Model E-38XW Thermosafe, Arlington Heights, IL) between two “dummy” containers filled with water to simulate shipping conditions. The shipping box was packed with temperature-stabilizing packs (PolarPack, SCA Thermosafe) according to American Red Cross standard procedures. The shipping box with PLTs was held at 20–24°C without agitation for 48 hours. The temperature was monitored with a thermocouple (placed between aliquots D and E) thermometer (Omega, Stamford, CT) with a 2-hour interval between each temperature recording. After the period without agitation, PLT aliquots D and E were returned to the flatbed reciprocal shaker (65–70 cycles/min.; Helmer Platelet Storage System, Helmer Labs Inc., Noblessville, IN) at 20–24°C. All other aliquots were continuously agitated on the same shaker.

### Drug delivery

All drugs were dissolved in DMSO. Final drug concentrations in the PLT aliquots were 1 µM and 50 µM for VX-702 and PD98059, respectively, which were identical to those previously reported [20], [19]. Based on results reported by Kuliopulos and colleagues, 1 µM VX-702 completely inhibits activation of p38 MAPK by thrombin, SFLLRN, AYPGKF, U46619, and collagen in PLT suspensions [20]. Final DMSO concentration in PLT aliquots B, C and E were 0.016% and 0.135% for VX-702 and PD98059, respectively.

### PLT assays

All PLT units were sampled by syringe (approximate volume of 3.8 mL) on Days 1, 4 and 7. PLT concentrations and mean PLT volume (MPV) were measured using a hematology analyzer (CellDyn 3700, Abbott Park, IL). PLT extracellular pH was measured using a bench top meter (Orion, Thermo Scientific, Beverly, MA) and pH electrode (Accu-pHast, Fisher Scientific, Pittsburg, PA). The extent of shape change (ESC) and hypotonic stress responses (HSR) were measured turbimetrically using a Chrono-Log SPA 2000 (Chrono-Log Corporation, Havertown, PA) as previously described [21]. PLT aggregation was measured using a lumiaggregometer (Chrono-Log) and 10 µM of ADP (Chrono-Log) with 10 µg/mL of collagen (Chrono-Log), as final concentrations. Aggregation was expressed relative to a maximum slope and amplitude. PLT morphology was assessed by phase microscopy as the percentage of PLTs with discoid morphology as previously described [12]. Oxygen, carbon dioxide, glucose, and lactate levels were determined using a blood gas analyzer (Cobas b221, Roche Diagnostics, Indianapolis, IN). The bicarbonate concentration was calculated automatically from pH and CO_2_ (37°C) levels. Rates for bicarbonate neutralization, glucose consumption and lactate generation were calculated from measurements on Day 1 and Day 7 and normalized by day and 10^10^ PLTs.

Flow cytometry (FACSCalibur, BD Biosciences, San Jose, CA) was utilized to measure the percent of PLTs expressing membrane-bound CD62P (p-selectin) and CD42b (GP1bα). PLT samples were diluted to 1×10^6^ PLTs/mL with phosphate buffered saline (PBS) (GIBCO, Invitrogen, Eugene, OR) supplemented with 0.1% human albumin (HA) (American Red Cross, Blood Services; Washington DC). Sample aliquots were incubated with fluorescein isothiocyanate (FITC)-conjugated CD61 (Biolegend; San Diego, CA) monoclonal antibodies and with phycoerythrin (PE)-conjugated CD62P (Biolegend) or PE-conjugated CD42b monoclonal antibodies (Biolegend) at saturating concentrations for 15 and 120 min, respectively, at 22°C. Mouse IgG_1_ PE and FITC PLT isotype control (Biolegend) were used at saturating concentrations as negative controls. Appropriate color compensation was set for FITC fluorescence and PE fluorescence by using 530 nm and 585 nm bandpass filters, respectively. PLTs were identified by their characteristic light scattering and binding of CD61.

Changes in PLT plasma membrane asymmetry were measured using Annexin V binding to phosphatidylserine (PS) translocated from inner to the outer leaflet of the plasma membrane. Samples were diluted to 1×10^6^ PLTs/mL with manufacturer supplied binding buffer and incubated with FITC-CD61 and PE-conjugated Annexin V (Becton Dickinson Immunocytometry Systems, San Jose, CA) according to the manufacturer's protocol. PLTs were gated by forward and side scattering and binding of CD61. The percent of PLTs which exhibited red fluorescence was measured by flow cytometry (FACSCalibur). Standard three-color beads (Calibrite™ 3, BD Biosciences) were used for daily instrument calibration and FITC and PE color compensation.

Changes in the mitochondrial membrane potential (MMP) were determined using a MitoProbe JC-1 Assay Kit (Molecular Probes, Invitrogen). Samples were diluted to 1×10^6^ PLTs/mL with PBS supplemented with 0.1% HA and stained with 1 µmol/L of JC-1 for 15 min at 37°C. Samples were analyzed using a flow cytometer (FACSCalibur).

Intracellular reactive oxygen species (ROS) generation was monitored by measuring changes in fluorescence resulting from the oxidation of intracellular probes. Dihydroethidium (DHE) (Molecular Probes, Invitrogen) was utilized as an intracellular superoxide indicator (ISOA). Samples were diluted to 1×10^6^ PLTs/mL with PBS supplemented with 0.1% HA and stained with 5 µmol/L DHE for 15 min at 37°C. Fluorescence was detected in the FL2 region of a FACSCalibur flow cytometer. Changes in intracellular hydrogen peroxide (IHP) production in PLTs were measured using 5-(and-6)-chloromethyl-2′,7′-dichlorodihydrofluorescein diacetate, acetyl ester (CM-H_2_DCFDA, Molecular Probes, Invitrogen). PLTs were diluted to 1×10^6^ PLTs/mL with PBS supplemented with 0.1% HA and stained with 5 µmol/L CM-H_2_DCFDA for 15 min at 37°C. The mean of green fluorescence (FL1 region) was measured using a FACSCalibur flow cytometer.

### Statistics

Determination of means and standard deviations of experimental values and Analysis of Variance with repeated measures were carried out by using standard software (Instat, GraphPad Software, San Diego, CA). A value of p≤0.001 was considered significant taking into account repeated measures for the 18 PLT assays utilized in this study and three testing days [22]. Statistical differences between paired values of Test-CA vs. Control-CA and Test-IA vs. Control-IA were determined by post Hoc tests with Bonferroni corrections. Outliers were identified by the extreme studentized deviate method (ESD, Grubb's method, GraphPad).

## Results

### Continuous Agitation

**Figure 1:**
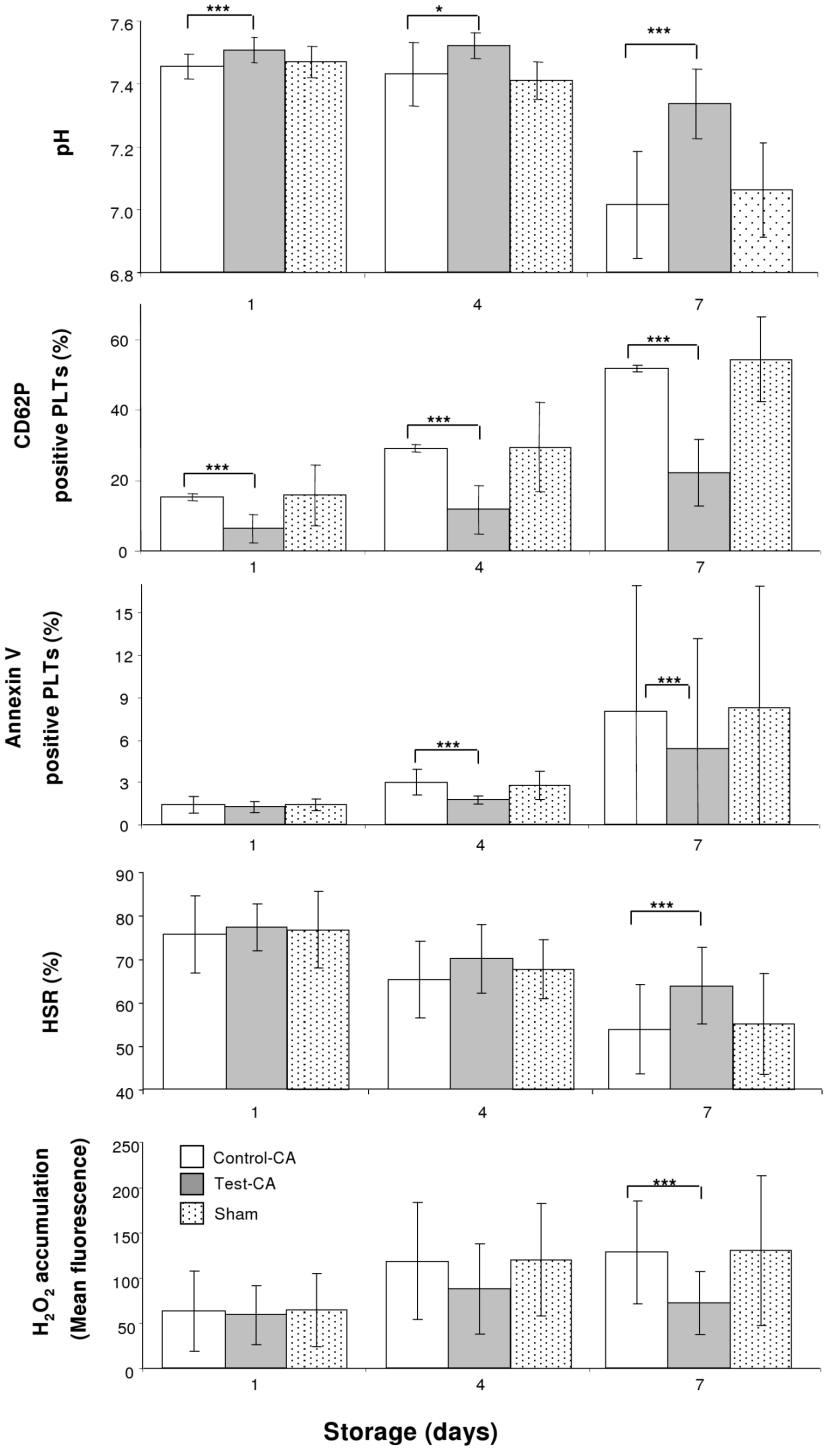
The pH levels, activation, Annexin V binding, HSR, and IHP accumulation of PLTs stored with continuous agitation. * - p<0.05; *** - p<0.001.

Results of the study utilizing VX-702 MAPK inhibitor for the continuous agitation arm are presented in Figure 1 and Table 1. PLT concentration values were unchanged during storage and did not differ between Test-CA and Control-CA. Levels of the metabolic PLT storage parameters, glucose and bicarbonate, were greater and lactate levels were less in Test-CA aliquots compared to those of Control-CA aliquots (Table 1). As a result, pH levels were greater in Test-CA compared to those of Control-CA (Figure 1). Carbon dioxide and oxygen levels were similar in all aliquots. Structural PLT storage parameters were assessed by measuring MPV, morphology, Annexin V binding, and expression of CD62P and CD42b. MPV values an the percentage of Annexin V positive PLTs (Figure 1) were less and morphology score was greater in Test-CA aliquots compared to those of Control-CA aliquots. PLT activation (Figure 1) was less and CD42b expression (Table 1) was greater in Test-CA aliquots compared to those of Control-CA aliquots. Functional PLT in vitro parameters were evaluated by measuring HSR, ESC and aggregation. The values of HSR and ESC were greater in Test-CA aliquots on Day 7 than those of Control-CA aliquots (Figure 1 and Table 1, respectively). Neither the slope nor the amplitude were different when comparing aggregation results from Control-CA and Test-CA aliquots (Table 1). Mitochondrial PLT in vitro function was assessed by MMP and accumulation of ISOA (Table 1) and IHP during storage (Figure 1). MMP values were greater and ROS accumulation was less in Test-CA compared to those of Control-CA aliquots. No DMSO toxicity was observed during 7 days of storage as Sham values of all parameters were comparable to those of Control-CA (Figure 1; Table 1, days 1 and 4, data not shown).

**Table 1:** PLT parameters assessed on Day 7 of storage with continuous and interrupted agitation with or without a MAPK inhibitor, VX-702.

Parameters	Control CA	Test CA	Sham	Control-IA	Test-IA
PLT Concentration (10^6^/mL)	1276±144	1284±142	1254±134	1224±146	1232±138
MPV (fL)	8.51±1.00	8.12±0.96‡	8.59±1.10	9.28±1.30	8.48±1.03††
Morphology (%)	52±12	63±14†	49±14	32±11	50±13†
CD42b (Mean Fluorescence)	410±74	484±74†	408±72	305±45	411±72††
ESC (%)	16.1±4.4	19.0±4.9†	15.8±5.1	11.4±4.1	13.6±4.5‡‡
Aggregation, Slope (%)	108.9±15.6	110.8±15.4	107.5±10.5	82.3±19.6	91.0±10.9
Aggregation, Amplitude (%)	90.6±8.5	88.3±8.1	89.6±6.2	73.6±19.4	77.6±8.9
Glucose (mM)	8.8±2.4	11.2±2.0†	8.8±2.2	6.5±2.6	8.1±2.5††
Lactate (mM)	20.4±3.3	15.8±2.9†	21.1±3.7	26.0±4.3	22.1±4.4††
Bicarbonate (mM)	5.5±1.6	8.3±1.2†	5.5±1.3	2.5±0.6	4.1±1.0††
pO_2_ (mm Hg)	151.6±9.9	149.2±8.6	151.7±8.3	164.9±6.5	156.8±6.4††
pCO_2_ (mm Hg)	23.3±2.7	22.5±2.1	23.9±2.6	19.8±2.4	19.8±1.4
MMP (%)	83.0±7.4	86.4±9.7‡ ∥	86.2±9.7	71.7±15.6	81.9±10.3††
ISOA (%)	25.0±3.7	20.7±3.9†	23.4±3.9	28.0±4.8	24.0±5.1††

‡ p<0.01,

† p<0.001, compared to that of Control-CA values.

∥ Calculated without outlier.

‡‡ p<0.01,

†† p<0.001, compared to that of Control-IA values.

Results of the study utilizing an ERK MAPK inhibitor, PD98059, with continuous agitation are presented in Table 2. In contrast to results of studies with p38 MAPK inhibitor, no differences were observed in any PLT properties stored in Control-CA and Test-CA aliquots. All in vitro parameters of Control-CA aliquots were in the range of normally stored PLTs and were comparable to those obtained from the Control-CA aliquot of the VX-702 study.

**Table 2:** PLT parameters assessed on Day 7 of storage with continuous and interrupted agitation with or without ERK MAPK inhibitor, PD98059.

Parameters	Control-CA	Test-CA	Sham	Control-IA	Test-IA
PLT Concentration (10^6^/mL)	1348±103	1323±97	1320±104	1265±142	1211±142
MPV (fL)	8.20±0.79	8.12±0.84	8.13±0.87	9.08±0.96	9.36±1.28
Morphology (%)	58±9	56±9	56±8	36±14	38±8
CD62P (%)	49.5±14.4	54.3±11.4	53.7±9.0	74.2±9.3	73.4±9.7
CD42b (Mean Fluorescence)	872±104	899±94	883±105	627±137	608±48
Annexin V (%)	8.3±4.7	10.5±3.5	9.8±2.8	27.0±11.8	31.3±16.8
ESC (%)	21.4±2.2	20.7±2.2	21.2±3.3	12.0±4.5	11.6±3.5
HSR (%)	62.6±13.3	63.6±11.2	65.2±9.8	39.1±18.5	37.6±12.8
Aggregation, Slope (%)	112.2±10.1	109.8±14.5	108.8±21.0	82.4±14.5	85.3±17.9
Aggregation, Amplitude (%)	91.8±10.0	85.7±6.6	84.4±7.4	61.8±27.2	65.1±18.4
Glucose (mM)	11.1±1.4	10.7±1.6	10.9±1.7	7.0±2.8	7.5±2.3
Lactate (mM)	16.0±1.6	15.0±1.6	15.2±1.2	23.8±4.1	22.2±3.2
Bicarbonate (mM)	8.0±0.8	8.4±0.6	8.5±0.7	3.0±1.3	3.3±1.3
pH	7.23±0.11	7.27±0.09	7.24±0.08	6.72±0.39	6.82±0.33
pO_2_ (mm Hg)	135.5±6.3	136.7±6.3	135.8±4.3	158.4±14.4	158.4±11.2
pCO_2_ (mm Hg)	26.2±2.0	25.9±1.5	27.2±2.3	19.9±2.9	19.9±3.9
MMP (%)	82.3±8.3	77.7±7.5	74.4±17.9	58.7±23.8	58.2±17.1
ISOA (%)	31.8±6.2	34.2±4.8	33.1±5.0	40.7±5.2	44.5±6.0
IHP (Mean Fluorescence)	764±751	761±845	797±752	1354±800	1024±494

### Interruption of Agitation

**Figure 2:**
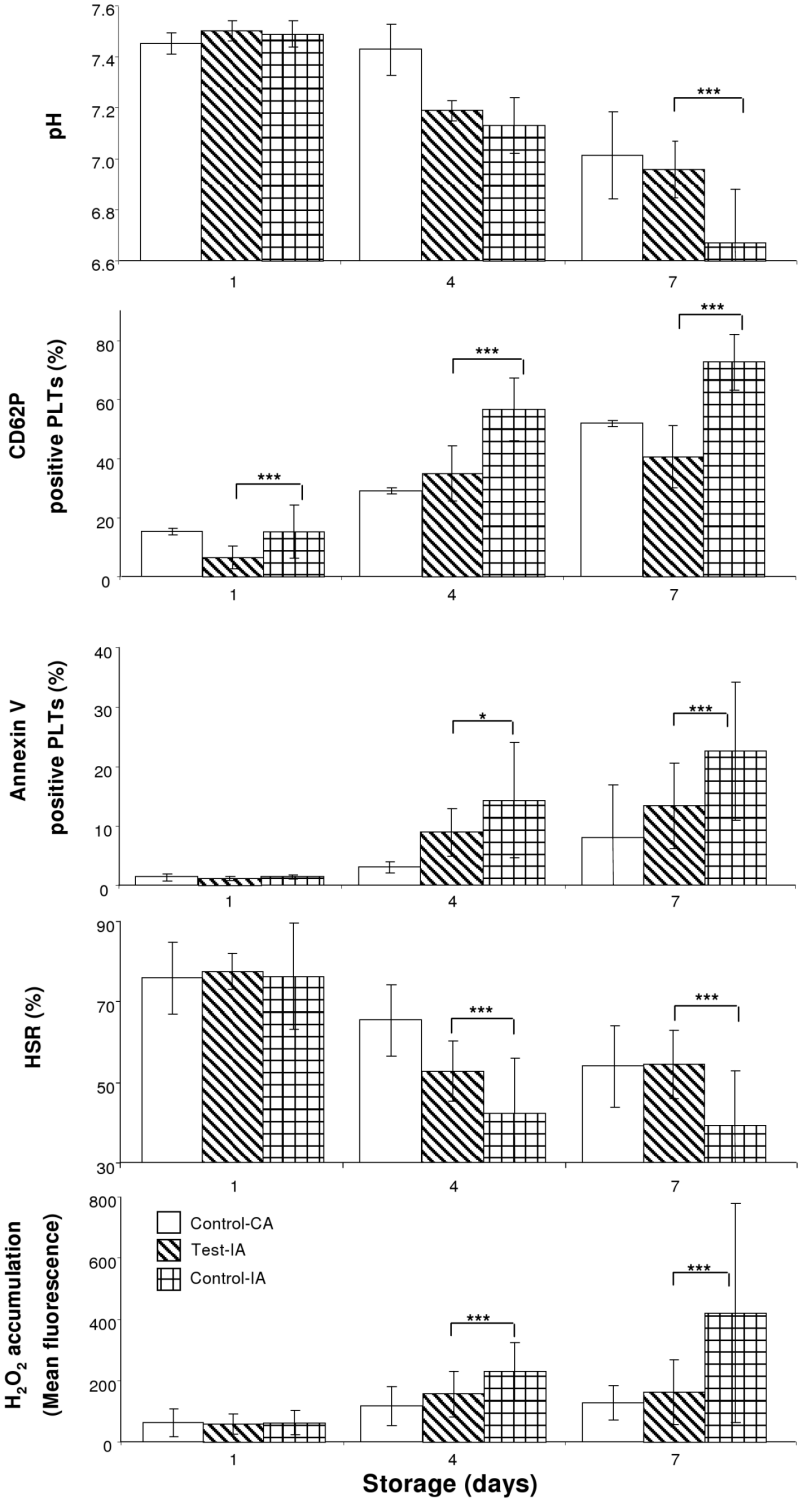
The pH levels, activation, Annexin V binding, HSR, and IHP accumulation of PLTs stored with 48 hours of interruption of agitation. * - p<0.05; *** - p<0.001.

Results of the study utilizing VX-702 MAPK inhibitor including an interruption of agitation are presented in Figures 2 and Table 1. PLT concentrations were similar in Control-IA and Test-IA aliquots. Glucose and bicarbonate levels were greater and lactate levels were less in Test-IA aliquots compared to those of Control-IA aliquots (Table 1). In contrast, lactate levels of Test-IA aliquots were similar to those of Control-CA aliquots (p>0.05). The pH levels (Figure 2) of Test-IA were greater than those of Control-IA but similar to those of Control-CA (p>0.05). The partial pressure of oxygen was less in Test-IA than that of Control-IA, but the partial pressure of carbon dioxide was similar for both aliquots (p>0.05). MPV values (Table 1) and levels of Annexin V binding (Figure 2) were less and morphology score was greater in Test-IA aliquots than those of Control-IA and were similar to those of Control-CA aliquots. The CD62P measurement of PLT activation (Figure 2) was less and the expression of CD42b was greater in Test-IA aliquots than that of Control-IA. The values of the functional parameters, ESC (Table 1) and HSR (Figure 2), were greater in Test-IA aliquots than those of Control-IA. HSR values of Test-IA were similar to those of Control-CA. In contrast to HSR and ESC results, no significant differences were observed in aggregation results between Control-IA and Test-IA (Table 2). The PLT MMP was greater and ROS accumulations (Figure 2 and Table 1, respectively) were less in Test-IA than those of Control-IA and were similar to those of Control-CA.

Results of the study utilizing an interruption of agitation with the ERK MAPK inhibitor, PD98059, are presented in Table 2. Similar to results with continuous agitation, addition of PD98059 in PLTs held without agitation for 48 hrs did not improve any in vitro PLT storage parameters relative to Control-IA. PLT storage parameters of Control-IA aliquots followed the same trends relative to Control-CA as previously described in the VX-702 interruption of agitation study.

## Discussion

PLT undergo a deteriorative process after collection known as the PLT storage lesion (PSL) which is characterized by structural, metabolical and functional in vitro decrements. Structural changes are manifested by declines in morphology, increases in mean PLT volume and activation, shedding of GP1bα and phosphatidylserine exposure. Metabolic decrements include increased glucose utilization with lactate accumulation and subsequent loss of bicarbonate buffering capacity, resulting in decreased pH levels. Functional changes in PLTs can be observed by decreased HSR and ESC with diminished PLT aggregation in response to single agonists [23]. The PSL is also characterized by reduced mitochondrial function resulting in the accumulation of ROS and loss of MMP [14].

During storage, PLTs lose their ability to aggregate using a single agonist and to adhere to the endothelial surface, most likely, due to structural changes to the vWF receptor complex which is cleaved by metalloproteinase 1. PLTs release metalloproteinases during storage, which cleave GP1bα, one of the units of the vWF receptor. Inhibition of metalloproteinase 1 (tumor necrosis factor-alpha-converting enzyme (TACE/ADAM17) by p38 MAPK inhibitor leads to improved PLT survival in a mouse model as well as decreased human PLT activation [15].

In our experiments with continuous PLT agitation, an inhibition of p38 MAPK by the second generation inhibitor, VX-702, improved all major storage parameters. Some of our data corroborate with previously published results. Thus, Canault and colleagues observed inhibition of GP1bα shedding when p38 MAPK inhibitor, SB203580, was used with mouse and human platelets whether the PSL was accelerated by increased temperature storage or by chemically induced oxidative damage of PLT mitochondria [16]. In addition, Schubert and colleagues demonstrated that the addition of SB203580 decreases both lactate production and PLT activation during storage [17]. Better maintenance of structural parameters may be a consequence of ubiquitous expression of inhibited p38 MAPK in many cellular compartments, normally able to phosphorylate a large number of different substrates [24]. It has been shown that p38 MAPK is distributed in the cytoplasm and regulates actin filament formation in murine embryos [25]. Moreover, activation of p38 MAPK resulted in F-actin reorganization in rat cardiomyocytes as well as in actin polymerization in mouse lungs [26], [27]. Improved maintenance of structural parameters by VX-702 during PLT storage (including MPV, morphology and percentage Annexin V positive PLTs) may also result in improved functional parameters such as HSR and ESC.

Another aspect of p38 MAPK inhibition by VX-702 is better maintenance of MMP and lesser ROS accumulation indicating better PLT mitochondrial function. It has been previously shown that ROS generated from mitochondria in cardiomyocytes during ischemia and reperfusion activates p38 MAPK and inhibition of p38 significantly prevented cell death arising from ischemia reperfusion [28]. PLT mitochondria are responsible for ATP production via aerobic respiration and for regulating cellular metabolism. Maintenance of PLT MMP ensures ATP production through oxidative phosphorylation which requires oxygen and electron transfer through the mitochondrial membrane. When mitochondria are damaged by oxidative stress, PLTs switch to anaerobic metabolism and begin to produce ATP through glycolysis. We observed decreased glycolysis and better preservation of most metabolic parameters due to the presence of VX-702.

Interruption of agitation affects the mitochondrial, metabolic, structural, and functional PLT parameters greater than those of normal storage. Inhibition of p38 MAPK by VX-702 reversed those changes in most in vitro parameters to levels observed in normally stored PLTs. For example, glycolysis rates were diminished greatly with the p38 MAPK inhibition and were comparable to those of Control-CA. Thus, glucose consumption rate for Test-IA (0.20±0.05 mM/day/10^10^ PLTs) and for Control-CA (0.19±0.05 mM/day/10^10^ PLTs) as well as lactate accumulation rate for Test-IA (0.52±0.09 mM/day/10^10^ PLTs) and Control-CA (0.44±0.12 mM/day/10^10^ PLTs) were not significantly different (p>0.05). These results corroborate previously published results describing the ability of p38 MAPK inhibitors to diminish glycolysis in working rat hearts, adipocytes, and murine skeletal muscles [29]–[31].

ROS generation in PLTs may activate additional kinases which are involved either in PLT metabolism or PLT functionality. For example, ERK MAPK is involved in thrombus formation. In contrast to results using p38 MAPK inhibitors, the inhibition of ERK did not influence PLT mitochondrial function or any structural, metabolic or functional parameters of PLT storage for either continuous or interrupted agitation. One explanation for this difference is that p38 MAPK is linked to the induction of pro-apoptotic signals when it is activated, and, unlike ERK MAPK, triggers a whole cascade of events [32]. There is accumulating evidence suggesting that p38 MAPK regulates a broad range of functional responses [32]. As a second generation p38 MAPK inhibitor, VX-702 was designed for greater affinity and greater selectivity for p38α MAPK than first generation inhibitors. Although its dissociation constant is not published, the parental compound, VX-745, exhibited a very clean selectivity profile [33]. Despite the fact that results with VX-702 are supportive in implicating the p38α MAPK pathway involvement in the PLT storage lesion, we cannot formally rule out that our observations with VX-702 are not the fortuitous result of some off-target binding to other kinases or signaling proteins.

Another pathway affecting PLT activation which can be induced by ROS involves phosphatidylinositol (PI) 3-kinase, known to affect GPIIbIIIa [34]. It has been reported that inhibition of PI-3 kinase diminishes ROS generation in neutrophils and macrophages [35]. Inhibition of PI-3-kinase also reduces PLT activation, diminishes glycolysis, and improves both PLT integrity and ESC values. However, enhancements to PLT storage were not maintained beyond Day 4 [36]. Inhibition of PI 3-kinase reduces PLT aggregation and does not improve morphology score [36], [37].

Based on our results, we speculate that inhibition of p38 MAPK improves the decrements in PLT mitochondrial function resulting from storage with continuous or interrupted agitation by inhibiting intracellular ROS activation of the kinase. Although PLT mitochondria are a source of ROS, cytoplasmic NADPH oxidase also can generate ROS [38]. It has been reported that excess ROS which escapes from mitochondrial redox system may activate NADPH oxidase toward increased production of ROS, resulting in what has been described as “a vicious circle” of oxidative stress [39]. Alternatively, NADPH oxidase can be directly stimulated by activation of p38 MAPK. Further studies are needed to clarify the sources of oxidative stress during normal storage and after prolonged period of IA.

In conclusion, inhibition of p38 MAPK, but not ERK, resulted in better maintenance of PLT mitochondrial, functional, structural and metabolic parameters during 7 day storage and restored PLT properties following an extended interruption of agitation to levels of continuously agitated PLTs. It is hoped that a better understanding of the pathways describing the PLT storage lesion may someday lead to the development of superior storage conditions for PLTs without pharmacological agents.
